# Molecular characterization and epidemic history of hepatitis C virus using core sequences of isolates from Central Province, Saudi Arabia

**DOI:** 10.1371/journal.pone.0184163

**Published:** 2017-09-01

**Authors:** Medhat K. Shier, James C. Iles, Mohammad S. El-Wetidy, Hebatallah H. Ali, Mohammad M. Al Qattan

**Affiliations:** 1 College of Medicine Research Center, King Saud University, Riyadh, Saudi Arabia; 2 Department of Medical Microbiology and Immunology, College of Medicine, Menoufia University, Menoufia, Egypt; 3 Faculty of Medicine, School of Public Health, Imperial College, London, United Kingdom; 4 Department of Surgery, College of Medicine, King Saud University, Riyadh, Saudi Arabia; National and Kapodistrian University of Athens, GREECE

## Abstract

The source of HCV transmission in Saudi Arabia is unknown. This study aimed to determine HCV genotypes in a representative sample of chronically infected patients in Saudi Arabia. All HCV isolates were genotyped and subtyped by sequencing of the HCV core region and 54 new HCV isolates were identified. Three sets of primers targeting the core region were used for both amplification and sequencing of all isolates resulting in a 326 bp fragment. Most HCV isolates were genotype 4 (85%), whereas only a few isolates were recognized as genotype 1 (15%). With the assistance of Genbank database and BLAST, subtyping results showed that most of genotype 4 isolates were 4d whereas most of genotype 1 isolates were 1b. Nucleotide conservation and variation rates of HCV core sequences showed that 4a and 1b have the highest levels of variation. Phylogenetic analysis of sequences by Maximum Likelihood and Bayesian Coalescent methods was used to explore the source of HCV transmission by investigating the relationship between Saudi Arabia and other countries in the Middle East and Africa. Coalescent analysis showed that transmissions of HCV from Egypt to Saudi Arabia are estimated to have occurred in three major clusters: 4d was introduced into the country before 1900, the major 4a clade’s MRCA was introduced between 1900 and 1920, and the remaining lineages were introduced between 1940 and 1960 from Egypt and Middle Africa. Results showed that no lineages seem to have crossed from Egypt to Saudi Arabia in the last 15 years. Finally, sequencing and characterization of new HCV isolates from Saudi Arabia will enrich the HCV database and help further studies related to treatment and management of the virus.

## Introduction

Hepatitis C virus (HCV) displays very high levels of genetic diversity which have been used to differentiate seven major genotypes and about 80 subtypes [1]. Although the different genotypes and subtypes share basic biological and pathogenic features, they differ in clinical outcomes, response to treatment and epidemiology [2]. The HCV genome is described to have an elevated percentage of sequence variation. When differences at the nucleotide level are between 31% and 33%, HCV strains are divided into genetically different genotypes. Differences between 20% and 25% specify different subtypes and variation below these levels indicate quasispecies [3]. Different HCV genotypes show a considerable variation in geographical distribution where genotypes 1, 2 and 3 have a worldwide distribution. “According to WHO report in 2000, it was found that HCV levels of infection in Egypt are the highest worldwide (15%) where HCV genotype 4 is the most predominant [4]. Unlike Egypt, only 1–3% of Saudi population are infected HCV from which 62% are HCV genotype 4 patients [5]. Genotype 5 is localized in South Africa, France, and Belgium, where, genotype 6 is found Indonesia, Thailand, and Singapore. [6].

One useful HCV region for subtype differentiation is the core that contains 573 nucleotides. This region contains higher mutation rate than the 5ʹ UTR region. The main function of the core protein is to form the capsid that protects HCV genomic RNA during viral infection. However, through interaction with many cellular proteins, HCV core protein also changes many host pathways [7]. The nonstructural 5B viral region (NS5B) contains 1777 bp and the sequence motifs are highly conserved among all the known RNA-dependent RNA polymerases (RdRps) [8]. RdRp is considered an important target for drug development [9,10], as well as the useful region for HCV genotypes and subtypes identification.

Studying evolution and genetic history of HCV genotype 4 is essential for several reasons. HCV genotype 4 responds less efficiently to interferon-based anti-HCV drug treatment than genotypes 2 and 3, especially in patients of African origin [11, 12]. Recently, the new treatment of HCV by Direct Acting Antiviral agents (DAA) provides pan-genotypic regimens that may be the most effective treatment of most of HCV patients [13, 14]. It has been hypothesized that this phenotype is the result of the long-term presence of genotypes 1 and 4 in Central African populations [12]. There are a number of query concerns about the origin and spread of HCV genotype 4 within Africa. Previous studies have not clearly demonstrated the current and past distribution of HCV genotype 4 strains among different countries or detect the geographic source of the HCV lineages present in the Middle East [12, 15–17]. In this study, we focus on the most common genotypes found in Saudi Arabia (genotype 1 and 4) and mainly in the central province including the capital Riyadh which is considered, from our point of view, an excellent environment for investigating the HCV epidemic history as the population size and number of infected patients are the most as reported before [18, 19]. Similar studies were done on NS5B region in the western province (Jeddah, Saudi Arabia) [20–22]. Our aim is to investigate a number of HCV isolates from Saudi Arabia, sequenced in the core region with analysis of nucleotide variation present. We also examined the epidemiological history of these isolates related to other neighbor countries.

## Materials and methods

### Ethics and consent statements

Subjects, including human material or human data, in addition to all written informed consents, have been obtained, documented and provided by Pathology Department, College of Medicine, King Saud University, Riyadh, Kingdom of Saudi Arabia (KSA). The project and data forms were approved by the Ethics Committee at College of Medicine and King Khalid University Hospital, King Saud University, Riyadh, KSA (Ref. no. 16/0172/IRB) in compliance with the Helsinki Declaration (https://www.wma.net/what-we-do/medical-ethics/declaration-of-helsinki/).

### HCV isolates

We used 95 serum samples from different HCV chronic patients, collected from King Khaled University Hospital (KKUH) according to ethical regulations. KKUH is a well representative model for this study as it is visited by most of HCV patients from the central province of Saudi Arabia which includes either male or female patients, patients of different ages, origins and from different locations including the capital Riyadh (where the higher population exist) or surrounding areas [23]. We used the same patients’ sera and data used previously to compare the 5’UTR genotyping results with those of HCV core [24]. The results of only 54 samples were used in this study because these samples gave a strong signal of PCR products. The unused 40 samples were not suitable for this study because of their lower viral titer, less than 50,000 copies/ml, where these samples gave no or bad results with either the universal primer of HCV 5’UTR region or HCV core primers despite they were positive to HCV antibodies. Few samples gave no results with any of the three primer sets detecting HCV core in either HCV genotype 4 or genotype 1 which prove that their genotyping results by 5’UTR (only genotype 1 or 4 were used) were incorrect. The samples in these mentioned cases were excluded. The viral load was detected using real-time PCR technique and COBAS AmpliPrep /COBAS TaqMan HCV Quantitative Test, version 2.0 Instrument (Roche Molecular Diagnostics, California, USA) and ranged from 200,000 to 25,000,000 copies / ml.

### HCV genotyping

The methods for viral RNA extraction, RT-PCR amplification, sequencing reaction and analysis of all HCV isolates have been described previously [24]. Both of HCV core and NS5B primers were designed at the conserved regions that would give the longest sequence and cover the most known genotype 4 and 1 sequences, according to references obtained from HCV database (http://hcv.lanl.gov/content/sequence/NEWALIGN/align.html). All sequences were aligned together by MEGA 6 software [25], http://www.megasoftware.net/, where primers locations were selected. Four primers were designed for amplification of HCV core region in which one forward primer (at position 426–446 relative to H77) works with either one of three reverse primers (at position 732–751 relative to H77). For HCV core primers (nt 426 and nt 751 related to H77), sequences are as follows: The forward primer (CF) 5’-CAG ATC GTT GGC GGA GTT TAC-3’, the reverse primer (CR) 5’- ATRTATCCCATGAGRTCGGC -3’ where sequences of NS5B primers (nt 7952 and nt 8617 related to H77) are as follows: The forward primer (NF) 5’ACCACATCARCTCCGTGTGG’3, the reverse primer (NR) 5’CTCGTCATRGCYTCCGTGAA’3. The products size of HCV core and NS5B are 326 bp and 684 bp, respectively, that is sufficient for variation detection in these regions. Purified PCR products were sequenced directly with Big Dye Terminator V 3.1 kit (Applied Biosystems, Foster City, CA) using GA-3130 DNA automated sequencer.

### Sequence collation

All available HCV genotype 4 sequences were downloaded from GenBank, National Center for Biotechnology Information (NCBI) [26]. Sequences were retained if extended over the sub-genomic region sequenced in this study (positions 426–751 relative to H77) and originated from the Middle East or Africa, resulting in 486 reference sequences. Sequences were combined with the new sequences gathered in this study and aligned by hand using AliView 1.17.1 [27]. Three alignments were created; all genotype 4 Saudi Arabian sequences (140 sequences), all genotype 1 Saudi Arabian sequences (54 sequences), and all gathered samples (473 sequences). For each isolate, two pieces of data were collected from online databases and the primary literature: year of sampling and country of origin. To study the geographic distribution, we used isolate sequences of Middle Eastern and African countries (S1 Table).

### Phylogenetic analysis

Each sequence was run through the HCV genotyping interface, using BLAST and HCV database, to obtain basic genotype information [28, 29]. This was used to place sequences in an alignment with reference genomes from the respective genotype. The table of confirmed HCV genotypes/ subtypes is available at https://talk.ictvonline.org/ictv_wikis/flaviviridae/w/sgflavi/35/table-1-confirmed-hcv-genotypessubtypes-may-2015. For HCV core trees, both genotype 1 and genotype 4, we used a maximum-likelihood phylogeny which was generated using Garli 0.951 [30]. We used a general time-reversible substitution model with estimated parameters, estimated base frequencies, no invariable sites and variable sites estimated with a gamma distribution with 4 rate categories. Confidence in the phylogeny was assessed by using 400 bootstrap replicates that were created and summarized with TreeAnnotator 1.8 [31]. Sequences were subtyped according to being placed in a clade with a subtype reference genome with bootstrap support > 0.7.

### Bayesian evolutionary analysis

We used the Bayesian Markov Chain Monte Carlo (MCMC) inference method implemented in BEAST v1.8 [31]. The epidemic history of each HCV alignment was estimated. Each analysis used an SDR06 nucleotide substitution model (two independent HKY+Γ substitution models–one for the first and second codon positions, and one for the third), a gamma model of among-site rate heterogeneity with 4 categories, and an uncorrelated log normal relaxed molecular clock. A Bayesian skyline coalescent model was used to estimate epidemic history, with 10 population groups. The effective population size for each group was given an uninformative uniform prior distribution, bounded at 0 and 10^100^. A normal prior distribution was placed upon the mean evolutionary rate parameter, with a mean of 7.21 x 10^−4^ and standard deviation of 1.6 x 10^−4^ chosen according to evolutionary rates seen in the core region in previous studies [32]. Each MCMC run contained 200 million states, sampled once every 5000 states; trees were sampled every 50,000 states. Multiple MCMC runs were calculated to ensure convergence and were combined to increase the accuracy of parameter estimates. Tracer software version 1.5, http://beast.bio.ed.ac.uk/tracer, was used to monitor MCMC convergence and effective sample sizes. Maximum clade credibility trees were calculated and annotated using TreeAnnotator 1.8 [31]. FigTree v1.3.1, http://beast.bio.ed.ac.uk/figtree, was used to color lineages according to their sampling location using the parsimony criterion.

## Results

### HCV genotypes and subtypes

**Fig 1:**
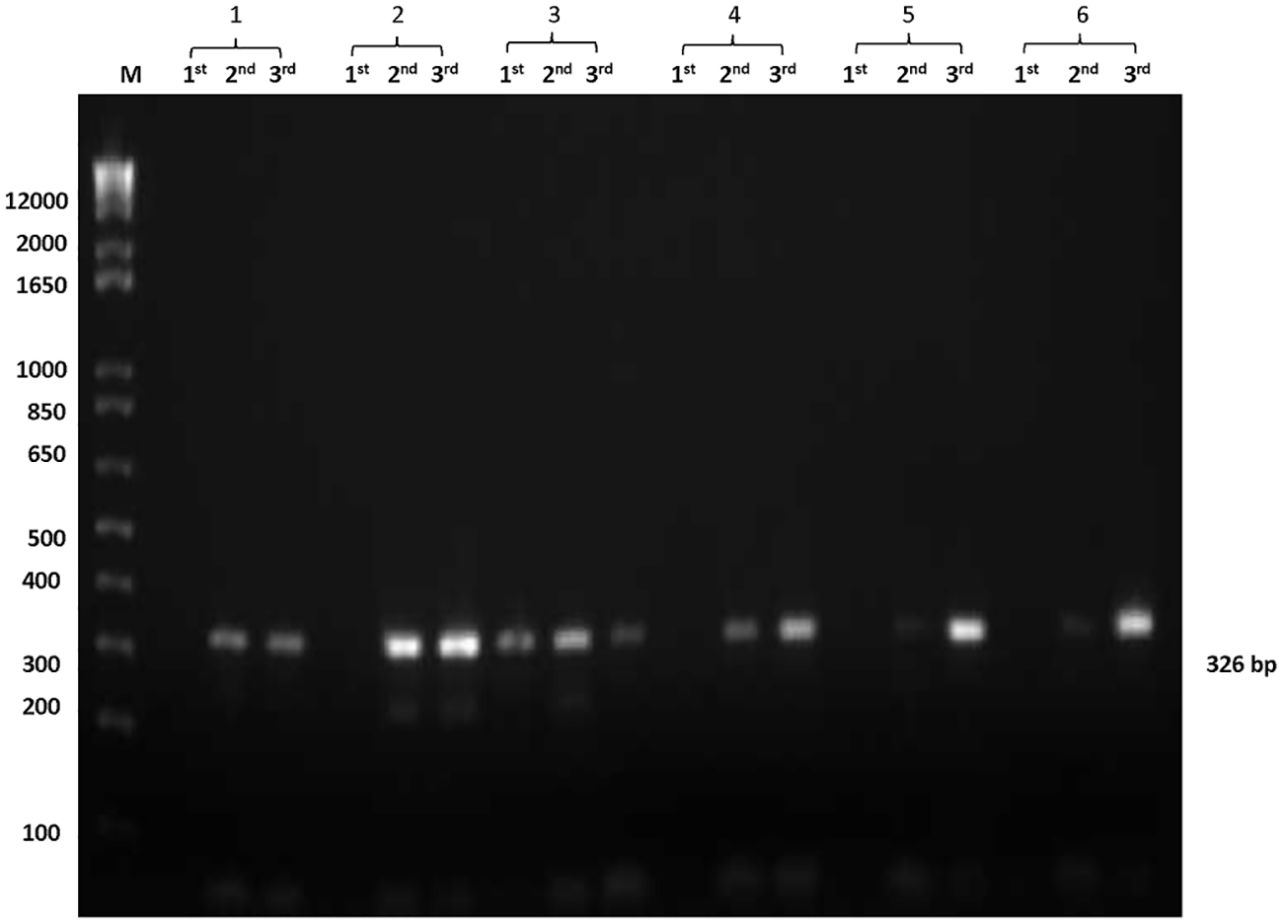
PCR products of HCV core region. PCR products were separated on 1.5% agarose gel. Three different primer sets were used to amplify 326 bp of HCV core region. The isolates appear in this gel were as follows: 1) EGSC1511 {4d}, 2) EGSC1512 {4d}, 3) EGSC1515 {4a}, 4) EGSC1520 {4d}, 5) EGSC1522 {4n} and 6) EGSC1556 {4d}.

For studying HCV sequences, we used serum samples from HCV chronic patients and extracted viral RNA, which was then subjected to direct sequencing. The results of 54 isolates showed that there were two major genotypes; 46 isolates were HCV genotype 4 and 8 isolates were genotype 1. Both genotypes produced PCR products with one or more of the three primer sets designed for HCV core region amplification (Fig 1).

All isolates that were used in this study have been investigated before by direct sequencing of HCV 5’UTR region [15]. Genotyping of HCV using core sequences revealed that some of the isolates have different HCV genotypes like EGSC1503, EGSC1525, EGSC1526, EGSC1543, EGSC1545, EGSC1547 and EGSC1552 that were genotyped as 4 previously while HCV core sequencing showed these isolates are genotype 1. Two other isolates, EGSC1519 and EGSC1521, couldn’t be genotyped accurately by 5’UTR sequencing only while core sequencing confirmed that these isolates are genotype 1 and 4, respectively.

Genotype identification of our HCV sequences, using blast search, relied on 95–99% and 85–93% identity for HCV core and NS5B regions, respectively. These identities were calculated against sequences of complete genome references according to subtype and country of origin [S2 Table]. These results were similar for both NCBI BLAST database and the specific HCV database indicating the same genotype. The nucleotide sequences of the HCV isolates have been deposited in the GenBank database under accession numbers from KU883372 to KU883417 for HCV core genotype 4 sequences, KU883418 to KU883425 for HCV core genotype 1 sequences and KU883426 to KU883436 for HCV NS5B genotype 4 sequences.

The HCV genotype 4 core isolates were subtyped into six main groups. 25 sequences were subtyped as 4d, while nine sequences were 4a and six sequences were 4r. Other subtypes such as 4m, 4n, and 4o divided the remaining six sequences equally. For HCV core genotype 1, two isolates were 1a and six were 1b. We picked nine isolates of those were genotype 4 by core sequences to be sequenced in NS5B region, which is known to be more reliable for subtyping according to higher variation rates, confirmed different subtypes resulted [33], to confirm subtyping results from HCV core. The Basic Local Alignment Search Tool (BLAST) search of the resulted sequences of both regions agreed in genotype classification, confirming all to be HCV genotype 4. The nine isolates selected for HCV NS5B sequencing showed similarity to subtypes detected by HCV core except for isolate EGSN1541 that was closer to 4a than 4d (data not shown).

### HCV nucleotide conservation and variation

Calculating the nucleotide variation percentage was done by hand and MEGA software for HCV isolates of each subtype separately. We calculated the percentages of variation and conservation between resulted sequences and reference sequences related to their estimated subtype and also we compared sequences of the same subtype to each other to estimate how far they are from each other or from consensus sequences. The references used were as follows: H77.NC_004102 (1a), EU781827 (1b), Y11604 (4a), DQ418786 (4d), FJ462433 (4m), FJ462441 (4n), FJ462440 (4o) and FJ462439 (4r) (Table 1). HCV core genotype 4 subtypes revealed that 4a is the most variable with 13% of nucleotides had variation in the same position of aligned sequences. For the most common subtype 4d, the variation was lesser than 4a; 9%. For HCV genotype 1 sequences, subtype 1a showed only 3% variation whereas subtype 1b showed 12% variation of the aligned nucleotides. Comparison of our isolates to all previously subtyped HCV sequences obtained from GenBank, shows high variation rate that reaches up to 8% for 4d samples, particularly isolate EGSC1504 and up to 7% for 4a samples, particularly isolate EGSC1536, where 4d and 4a represent most of the isolates used in this study (Table 1).

**Table 1:** HCV genotypes and subtypes compared in respect to nucleotides conservation and variation rates.

	HCV core genotype 1		HCV core genotype 4					
**Subtypes**	1a	1b	4a	4d	4m	4n	4o	4r
**No. of isolates**	2	6	9	25	2	2	2	6
**Total**	8		46					
**Conserved nucleotides**	277 (97%)	250 (88%)	247 (87%)	259 (91%)	279 (98%)	281 (98.5%)	270 (95%)	275 (96.5%)
**Varied nucleotides**	8 (3%)	35 (12%)	38 (13%)	26 (9%)	6 (2%)	4 (1.5%)	15 (5%)	10 (3.5%)
**Variation to HCV database References**	1–3%	2–4%	1–7%	0–8%	1–3%	0–5%	2–6%	1–4%
**No. of HCV database References used**	44	70	81	43	5	8	4	7
**Isolate with highest variation rate**	EGSC1543	EGSC1526	EGSC1536	EGSC1504	EGSC1509 EGSC1521	EGSC1522	EGSC1524	EGSC1531
**Total**	285 (100%)							

**Fig 2:**
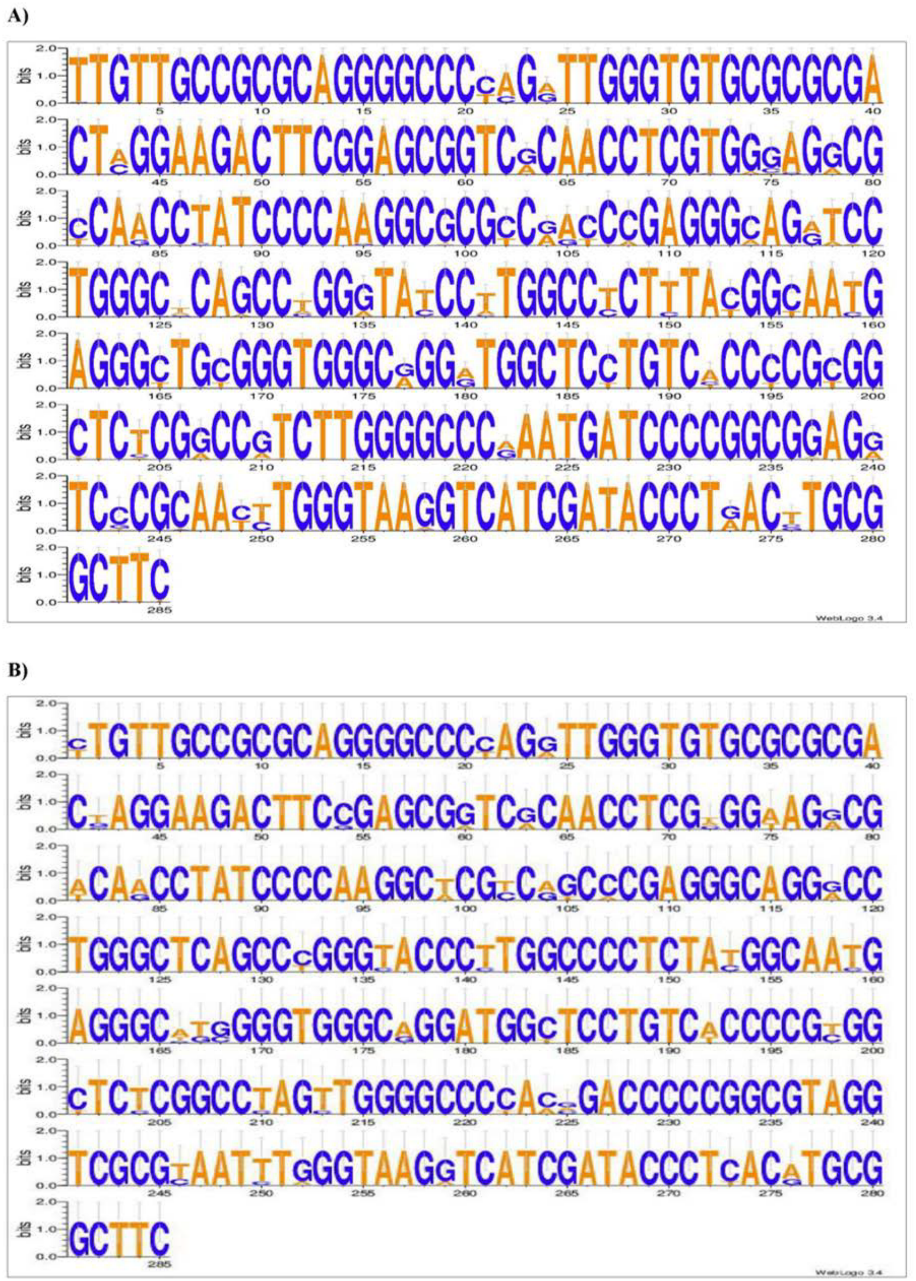
Sequence logo diagram of HCV core sequences. This was created by the alignment tool MEGA 5.05 Software and the WebLogo3 software (http://weblogo.threeplusone.com/create.cgi). The primers’ sequences at both ends were deleted from all represented sequences. The vertical bar is 2 bits high. The nucleotides overlap persistent in one position indicates the polymorphic sites. The height of each letter represents the relative proportion of each nucleotide at that position. (A) HCV core genotype 4 logo using forty-six isolates. (B) HCV core genotype 1 logo using eight isolates.

**Fig 3:**
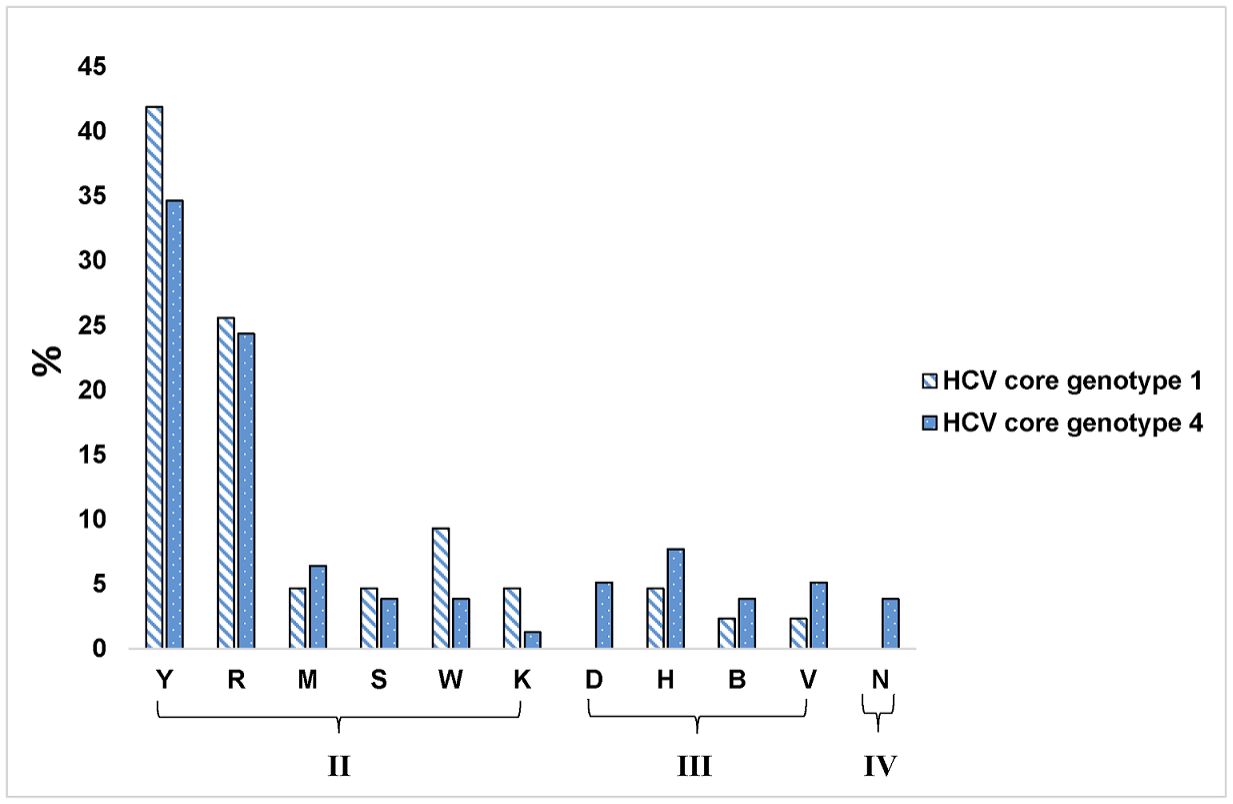
Bar graph representation of the nucleotide variation, expressed as mixed bases, among HCV genotype 1 and 4 sequences in the core region. The Greek numbers indicate how many nucleotides are called for the same positions where II, III, and IV indicates two, three and four substitutions, respectively, for the same nucleotide position. For the outline of the mixed bases, refer to Table 2.

When examining the HCV core genotype 4 isolates sequence logo, it was noticeable that variation among different subtypes occurred mostly between two nucleotides in 58 sites, while triplet variation occurred in 17 sites and only 3 sites showed 4 nucleotide variation (Fig 2). The double variation revealed that most of the variations are either pyrimidines (Y = C or T) in 27 nucleotide positions or purines (R = A or G) in 19 positions. Similar results occurred for HCV core genotype 1 and HCV NS5B genotype 4. These results demonstrated that core sequences of genotype 1 were the most conserved as only 34 out 285 sites (~12%) had variation compared to those of genotype 4 with 78 sites (~27%) showed variation (Fig 3) and (Table 2).

**Table 2:** Types and rates of nucleotides substitution among HCV genotypes 1 and 4.

Nucleotides substitution type	Mixed bases symbols*	HCV genotype 1 core		HCV genotype 4 core	
		No.	%	No.	%
II	Y	18	41.86	27	34.615
	R	11	25.581	19	24.359
	M	2	4.6512	5	6.4103
	S	2	4.6512	3	3.8462
	W	4	9.3023	3	3.8462
	K	2	4.6512	1	1.2821
III	D	0	0	4	5.1282
	H	2	4.6512	6	7.6923
	B	1	2.3256	3	3.8462
	V	1	2.3256	4	5.1282
IV	N	0	0	3	3.8462
	Total	34	100	78	100

* **M**: A or C, **R**: A or G, **W**: A or T, **S**: C or G, **Y**: C or T, **K**: G or T, **V**: A or C, or G, **H**: A or C or T, **D**: A or G or T, **B**: C or G or T and **N**: any base (*Cornish-Bowden (1985) Nucl*. *Acids Res*. *13*: *3021–3030)*.

### Phylogenetic trees

**Fig 4:**
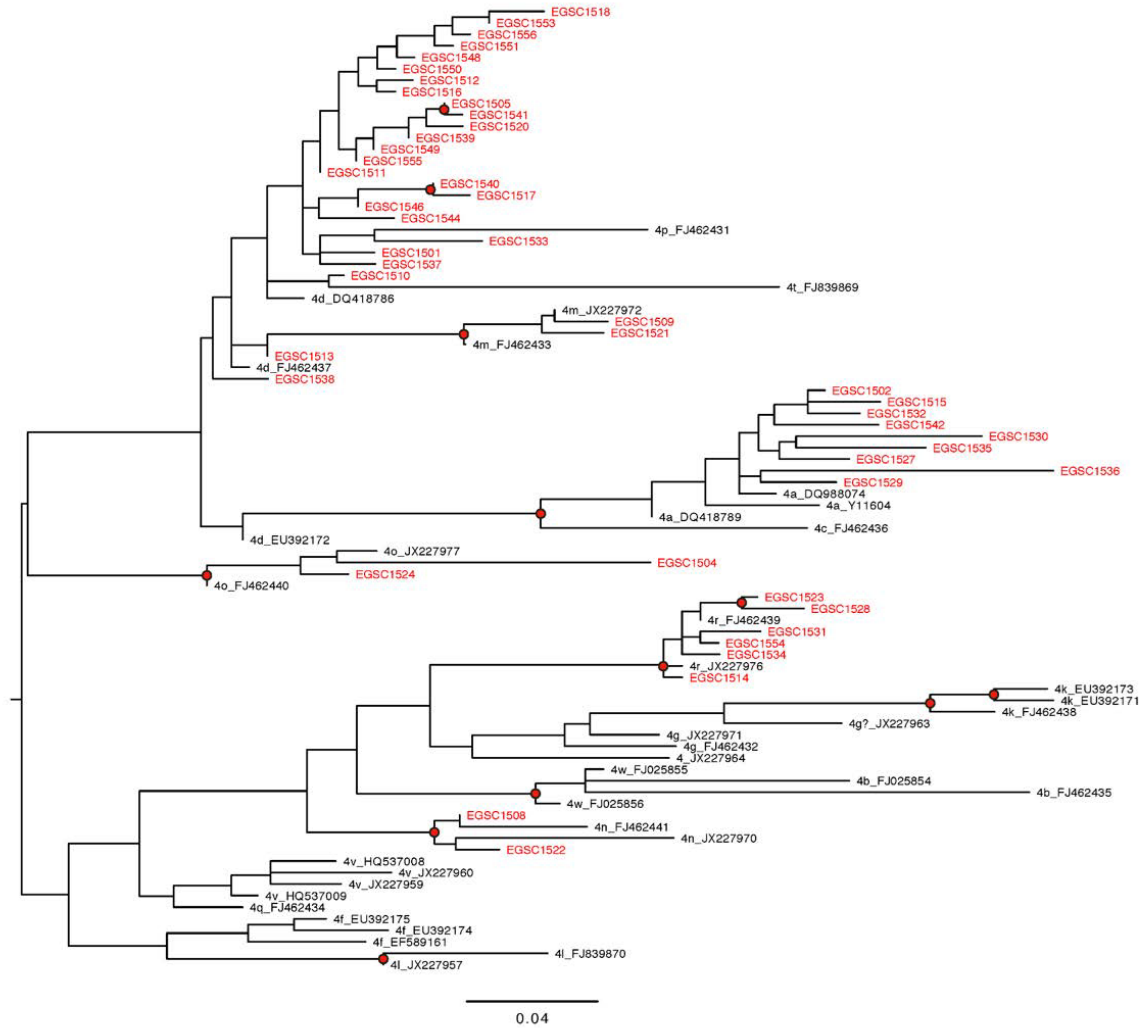
Maximum-Likelihood phylogeny of HCV core genotype 4 sequences. Maximum-Likelihood phylogenetic trees were used to subtype HCV core samples gathered in this study. Phylogenies were estimated from a 326-nt long alignment of the HCV core gene using a Generalized Time Reversible (GTR) substitution model with a gamma distribution model of among-site rate variation. Internal nodes are highlighted with a black circle if they have bootstrap support >70% (400 maximum likelihood replicates). Scale bar shows the expected number of substitutions per site.

**Fig 5:**
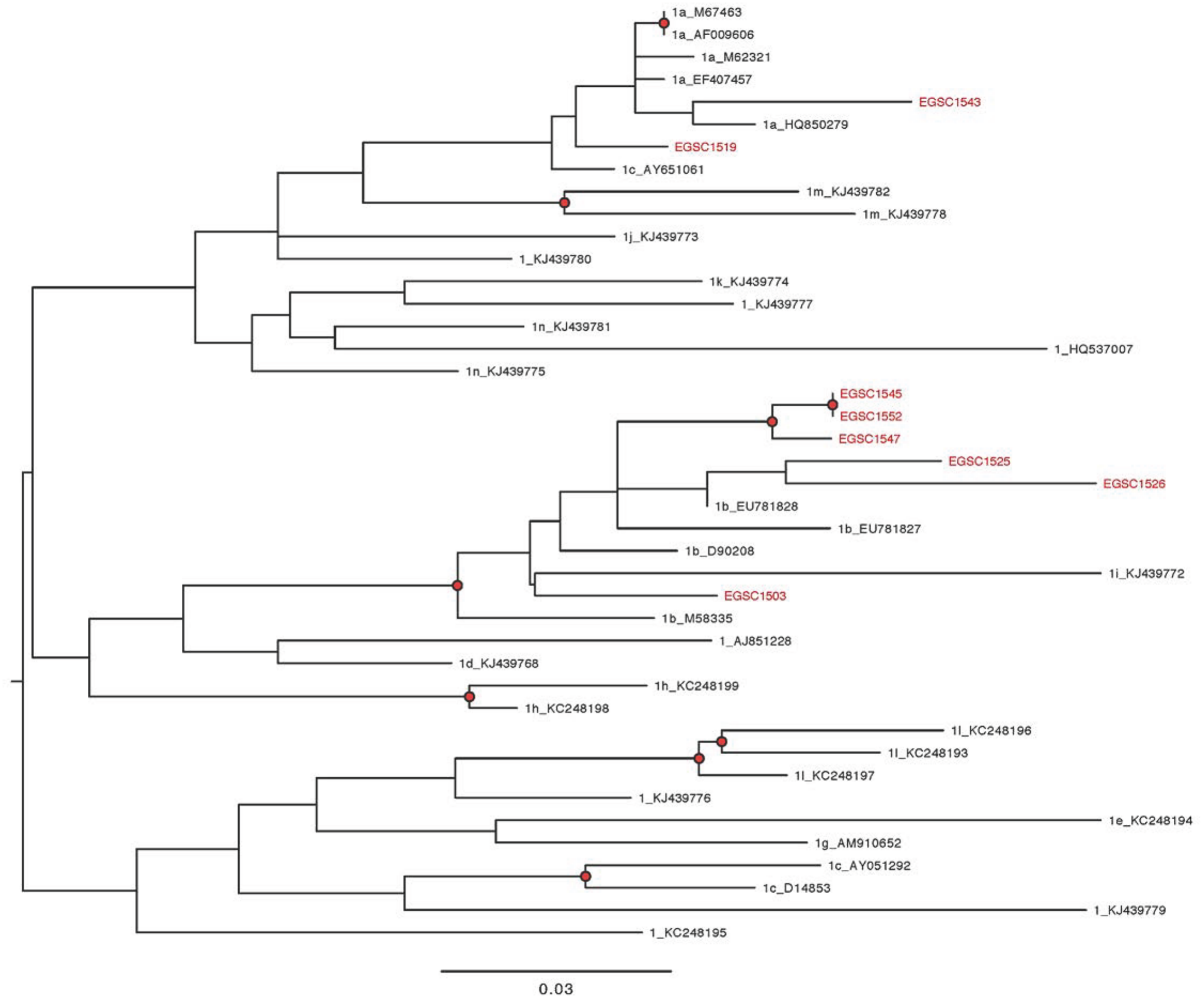
Maximum-Likelihood phylogeny of HCV core genotype 1 sequences. The phylogenetic tree, for the ten HCV genotype 1 sequences from HCV core region, was constructed in the same manner like that of HCV genotype 4.

The phylogenetic analysis of HCV core genotype 4 isolates showed the same genotype and subtype identity as BLAST and HCV database. Pairwise nucleotide alignment divided sequences into six different subtypes. The majority of isolates (n = 25) were grouped as one cluster with higher bootstrap values to references from the USA and Canada that were identified as 4d and lower values to references of 4t and 4p. Two isolates came closer to 4m references FJ462433 and JX227972 from Canada and UK, respectively. Nine isolates were gathered in subtype 4a cluster with references Y11604 and DQ988074 from Egypt and DQ418789 from USA. Two sequences showed higher bootstrap values to subtype 4o references JX227977 and FJ462440 from UK and Canada, respectively. Two other clusters of subtype 4r with 6 isolates and subtype 4n with two isolates showed up at the bottom of the phylogenetic tree with corresponding references. Other subtypes of genotype 4 didn’t pairwise with any of our sequences (Fig 4). Sequences thought to be HCV genotype 1 by BLAST were confirmed by phylogenetic analysis. Six sequences gave pairwise alignment with 1b references which are mainly from Europe and North America whereas only two sequences matched 1a references from USA (Fig 5).

### Recent transmission in Saudi Arabia

**Fig 6:**
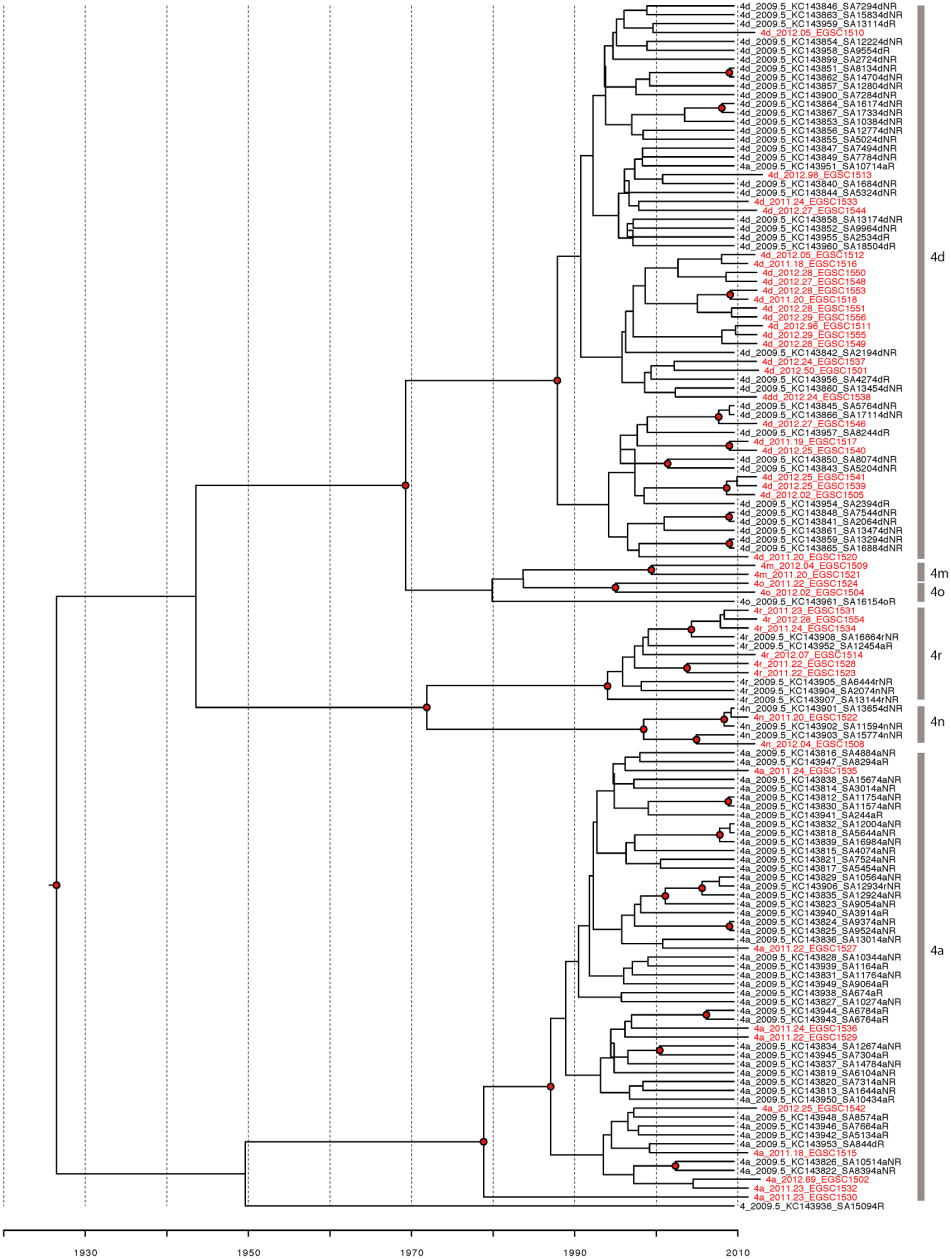
Maximum clade credibility molecular clock phylogenies of HCV genotype 4 isolates circulating in Saudi Arabia. Phylogenies were estimated from the combined alignments of all genotype 4 samples. Trees were generated using BEAST 1.8 and summarized using TreeAnnotator (http://beast.bio.ed.ac.uk). Branch lengths represent time-see scale bar at the bottom. Sequences originating in this study are colored red and labeled with their subtype, collection date and isolate name; other sequences are labeled with their subtype, collection date, accession number and isolate name.

**Fig 7:**
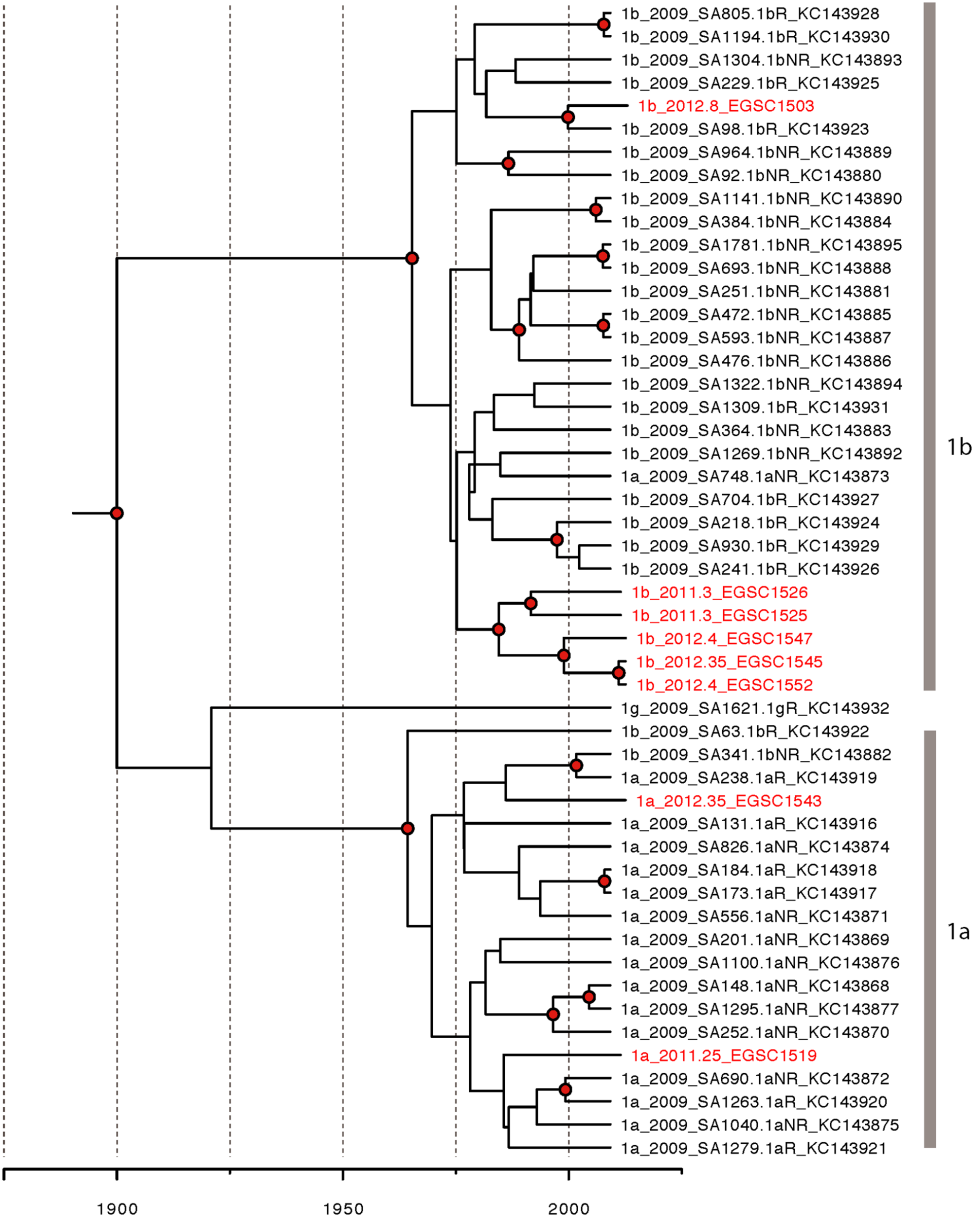
Maximum clade credibility molecular clock phylogenies of HCV genotype 1 isolates circulating in Saudi Arabia. The tree construction is similar to that of HCV genotype 4.

The maximum-clade credibility tree was generated from all genotype 4 and genotype 1 samples gathered in Saudi Arabia (Figs 6 and 7). The samples gathered in this study are dispersed across each tree, and aside from one clade in genotype 1b and one in 4d, do not seem to form clusters of recent transmission. Many previous studies used a closer number of samples to be used in phylogenetic analysis [34] therefore, the samples found in this study are likely a representative sample of HCV strains in Saudi Arabia.

**Fig 8:**
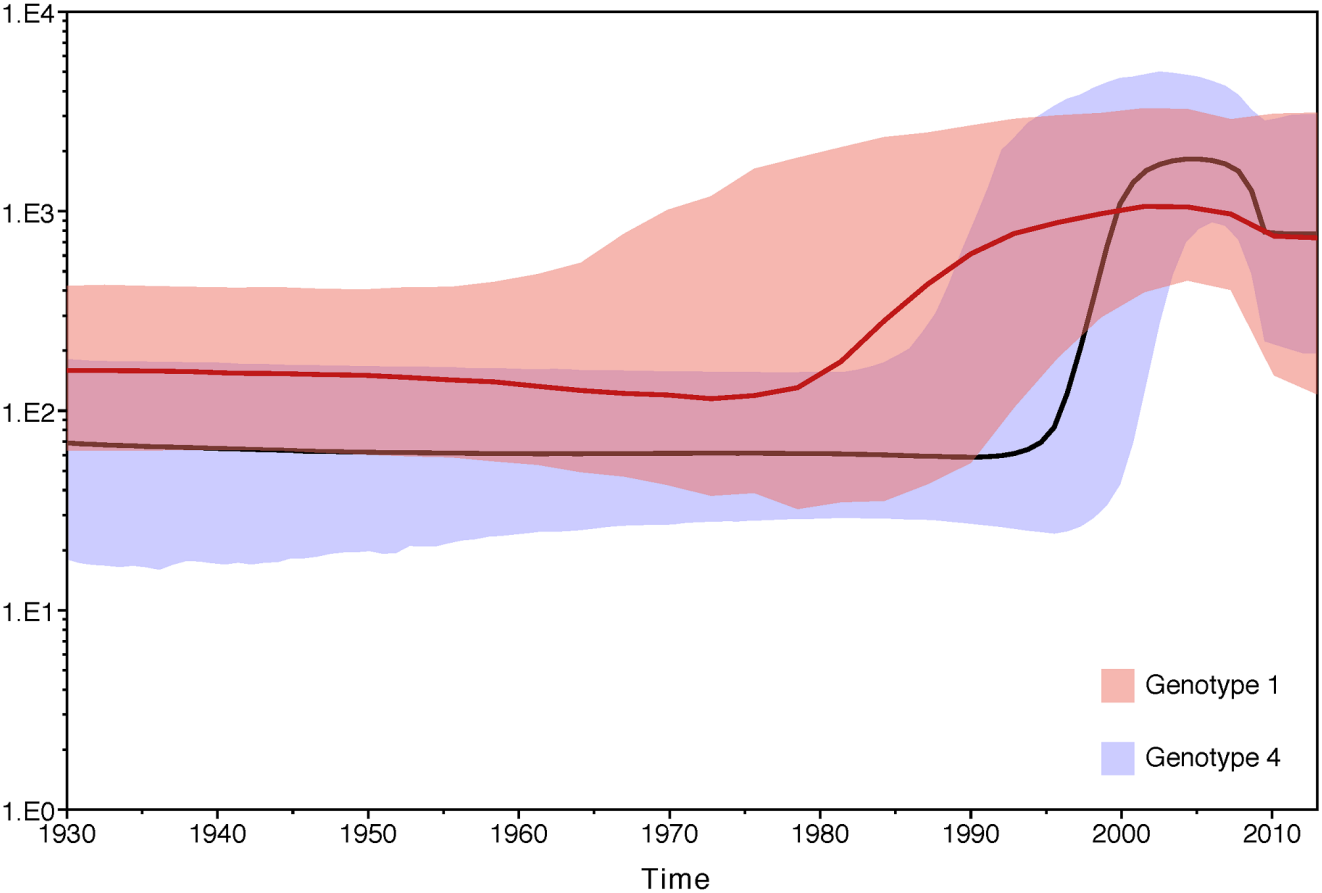
Transmission history and citation of HCV isolates in Saudi Arabia. HCV genotypes 1 and 4 in Saudi Arabia were analyzed by Bayesian Evolutionary Analysis, skyline plot. Each plot shows the change through time in epidemic size (effective population size x generation time) on a log scale. Thick lines indicate the median estimate and the shaded areas show the 95% highest posterior density credible region. The red curve was estimated from HCV genotype 1 sequences, while the blue curve was estimated from HCV genotype 4 sequences.

Previous study by The Bayesian skyline plot (BSP) analysis showed that HCV subtype 4d, which dominates the current HCV infections in Saudi Arabia since ~1970, was found since 1900 and increases slowly till 1950 then rapidly till 1980 [16]. The skyline plot produced by these analysis, colored according to genotype, shows how the virus’ epidemic size (calculated as effective population size x generation time) has changed over time (Fig 8), and seems to be divided into three distinct time periods: before 1960, when the virus circulated at a low level; between 1960 and 2005, where the epidemic size grew by two orders of magnitude; and the time between 2005 and the present, when the epidemic size fell by an order of magnitude. This pattern is more clearly seen in the genotype 4 skyline, likely due to the greater number of sequences used in that analysis.

This pattern is in contrast to the dynamic seen in Egypt and the Democratic Republic of the Congo, where the epidemic size stayed at the level reached by the exponential growth period or even rose intermittently [32, 35, 36]. This implies that measures to control the HCV epidemic have been more effective in Saudi Arabia than in those other countries, although HCV transmission seems to have reached an equilibrium resulting in an epidemic size of 5 x 10^2^.

### Sources of transmission into Saudi Arabia

**Fig 9:**
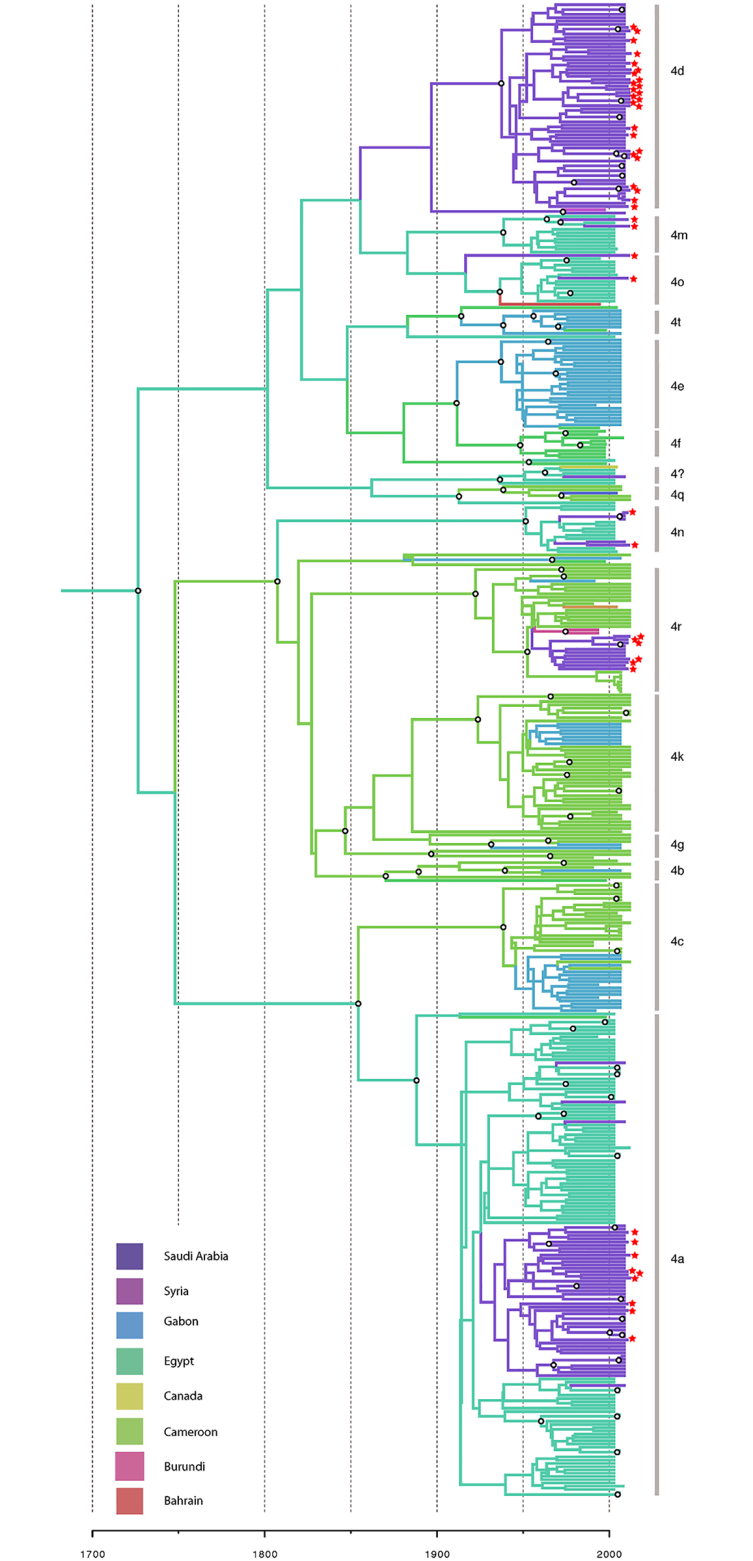
Comparison of HCV isolates sequences with those from African and Middle Eastern countries. The Maximum Clade Credibility molecular clock phylogenies were used to cite the HCV isolates from Saudi Arabia among isolates from the surroundings countries. Phylogeny was estimated from the alignment of genotype 4 sequences collected in this study as well as sequences from online databases. Tips are colored according to positions of sequence origin (see key). The locations of internal branches were deduced using parsimony and are colored similarly. Branch lengths represent time–see scale bar at the bottom. Nodes with a posterior probability >0.7 are labeled with a white circle. Samples gathered in this study are labeled with red stars; subtypes are indicated on the right side of the diagram.

To determine if a recent transmission from other countries was the source of recent epidemics in Saudi Arabia, we analyzed an alignment containing all genotype 4 samples gathered across Africa and the Middle East. The maximum-clade credibility tree, resulting from this analysis, was created with each branch colored according to its most probable country of origin (Fig 9). Strains associated with Saudi Arabia fall into two categories; clades that transferred into the country more than 50 years ago and have spread within the country ever since (e.g. 4d, 4r, the larger clade in 4a) and individual strains transmitted into the country over the past 50 years. While the former strains have both a central African and Egyptian origin, the latter strains all originate from Egypt. Even the most recent introduction of HCV from Egypt to Saudi Arabia had a coalescence time of 1974 (95% highest posterior distribution: 1966, 2002), implying that cross-border transmission makes up a very small proportion of HCV replication within Saudi Arabia in the modern day. Transmissions of HCV from Egypt to Saudi Arabia are estimated to have occurred in three major clusters: 4d was introduced into the country before 1900, the major 4a clade’s MRCA was introduced between 1900 and 1920, and the remaining lineages were introduced between 1940 and 1960. This suggests that the majority of HCV replicating in Saudi Arabia belongs to lineages that have been present in the country for half a century or more, and that modern migration presents a comparatively small source of HCV diversity. Most of the HCV transmission from other countries to Saudi Arabia originate in Egypt, but this is a relatively small number compared to the large quantity of HCV transmission occurring within the country. In fact, there are no lineages in the tree that seem to have crossed from Egypt to Saudi Arabia in the last 15 years, and in that time 40 branches were created within Saudi Arabia.

## Discussion

The overall HCV prevalence in Saudi Arabia reflects how the disease burden is difficult to be controlled without remarkable changes in the available treatment paradigm [37]. HCV was the seventh (95% UI, Uncertainty Interval) leading cause of death worldwide in 2013, compared with tenth in 1990 as reported by WHO [38]. DAAs that proved more effective and less toxic on genotype 1, however, more studies are needed for genotype 4 patients [39]. HCV genotyping is essential for identifying different types of HCV and thus determining the best approach for treatment. HCV genotyping can be done either by Real Time PCR assay of 5’UTR or by direct sequencing of different HCV viral regions. Genotyping that depends on a targeting 5’UTR is not the most accurate due to lack of nucleotide polymorphisms in 5’UTR [40, 41]. Direct sequencing analysis is considered the most accurate method. Both HCV core and NS5B viral regions are considered the most reliable regions for genotyping because of sufficient genetic diversity and absence of recombination events [42]. Genotyping of HCV by BLAST analysis depends on the comparison of newly obtained sequences to previously genotyped sequences in the HCV database relying on the percentage of identities among sequences. Continuous submitting of more genotyped sequences to database reinforce the usage of BLAST analysis as a more reliable method [43].

Mutation rate defines the ability of a virus to maintain important information while surviving with different environmental challenges [44, 45]. Previous studies showed the manifestation of more variability in sequences of HCV NS5B in comparison to the core sequences [46, 47]. The HCV core region has been proved to be better for HCV subtyping due to subtype-specific variation in a small proportion of sites [47, 48]. The use of the more variable region, NS5B, can be relied upon for accurate discrimination between and within subtypes [49]. Although NS5B is the preferred region for subtyping, amplification of this region is not always possible due to primer-target mismatch within the highly variable NS5B sequences. Unsuccessful sequencing of the NS5B gene has been documented by some investigators despite multiple attempts to amplify cDNA with the NS5B primers [50]. In the present study, three primer sets were used to amplify HCV core region but NS5B amplification required the use of seven sets.

Our HCV core genotype 1 and 4 sequences showed conservation rates of 94% and 73%, respectively. The high percentage of NS5B protein variation causes the RNA polymerase enzyme to make significant errors that result in nucleotide changes introduced in the viral genome [51, 52]. Previous studies indicated that variation in other viruses like HIV or HBV can enhance drug resistance variants development [53–55], similar to the development of HCV protease inhibitor resistance changes [56].

Since the sequence similarities in the core and NS5B regions between tested isolates and strains from the HCV database were >93 and 87.8%, respectively [57, 58], genotypes of sequences at the top BLAST outputs were considered as identical to the tested isolates. Based on the BLAST result, genotypes identified by core and NS5B sequence analysis were compared and our results showed that genotyping of both regions were identical. HCV core genotype 4 sequences of 46 isolates and the reference strains of genotypes 4a, 4d, 4m, 4n, 4o, and 4r were grouped into six clusters in the phylogenetic tree. The bootstrap values of each cluster exceeded 70%, indicating that the topology of core sequences was highly reliable. Those infected with HCV genotype 4 are likely to be Middle Eastern or South Asian, however, some references from USA, Canada, and Europe show some combination with our isolates especially 4d sequences. Only eight isolates were identified as HCV genotype 1. Those sequences were compared to different references representing different genotype 1 subtypes. Similar to genotype 4, Phylogenetic tree results of genotype 1 confirm that the two clusters, 1a and 1b, occurred and indicated Middle Eastern population.

Sequences of HCV core were compared to all published sequences from Saudi Arabia and Egypt because of more traveling incidents between the two neighbor countries. Egypt has a uniquely high HCV prevalence, resulting from collective campaigns to treat bilharziasis with injection therapy during the period from 1960 to 1987 [59]. Genetic analysis suggests that HCV was present at low prevalence in Egypt around the 1920s [35]. The majority of the Egyptian epidemic arrived from Central Africa in time between 1860 and 1925 (the estimated date of the MRCA of subtype 4a). It is clear that the Egyptian HCV epidemic arose from more than one Central Africa strain, as subtypes 4a, 4m, and 4o each transferred to Egypt separately [60]. A previous study of isolates collected in Kinshasa, the capital of the DRC during 2007, show that there was a lengthy period of endemic transmission, during which the effective number of infections was low, prior to the twentieth century. There was a severe switch from endemic infection to rapid exponential growth starting about the 1950s. The molecular clock and phylogeographic results clearly indicated that HCV genotype 4 epidemics in the Middle East, and North Africa, all originated from Central Africa [15].

The possibility of infection routes other than blood transmission including previous surgeries and dental procedures were well-documented. Contact-associated transmissions, such as toothbrushes, tattooing, folk body piercing, and non-medicalized circumcision, as well as exposure to skin lesion exude, are also significant risk factors [61]. Sharing of equipment used for parenteral practices is a significant way of HCV transmission in rural regions of Africa [62]. However, such methods as body scarification, clitodectomy using unsterilized tools, circumcision, and shaving by local barbers are known to be accountable for HCV transmission in Africa. Previously studied strains showed similarity with Asian, European and American strains, potentially due to migration events. That study reports a large community of South Asians settled in European countries [49]. This indicates that migration from one place to another might contribute to the distribution of HCV subtypes that would not, otherwise, exist in Saudi Arabia.

Saudi Arabia is an open community that embraces different cultures. In our study, only 16% of isolates were from non-African/Middle Eastern subtypes. Indeed, the coalescent tree shows evidence that the great majority of HCV infections within Saudi Arabia are acquired from and passed on to Saudi Arabian residents. Despite the different non-hygienic practices that cause the spread of different HCV subtypes in Saudi Arabia, the improvement of health services, investigation, and treatment that emerged in the last fifteen years have decreased the rate of transmission. The newly identified and published HCV sequences will enrich the online sequence databases which will increase the possibilities for more precise analysis and identification for any new HCV transmission to Saudi Arabia.

## Supporting information

S1 Table Geographic origin of sequences used in phylogenetic analysis. Number of sequences African and Middle Eastern countries that were used in the MCC tree of HCV core isolates from Saudi Arabia.
(DOCX)

S2 Table List of references used in the construction of maximum likelihood trees and phylogenetic analysis of both HCV Core region. All references express HCV complete genomes and were chosen according to the subtype and country of origin.
(DOCX)
