# Supplementary material for: Molecular characterization and epidemic history of hepatitis C virus using core sequences of isolates from Central Province, Saudi Arabia
Source: PLoS One. 2017 Sep 1;12(9):e0184163. doi: 10.1371/journal.pone.0184163 (PMC5580995; doi:10.1371/journal.pone.0184163)
Supplement: S1 Table — Number of sequences African and Middle Eastern countries that were used in the MCC tree of HCV core isolates from Saudi Arabia. (DOCX) [file pone.0184163.s001.docx]

| **Country** | **Sequences** |
| --- | --- |
| Bahrain | 1 |
| Burundi | 1 |
| Democratic Republic of the Congo | 101 |
| Cameroon | 16 |
| Egypt | 147 |
| Gabon | 62 |
| Rwanda | 1 |
| Saudi Arabia | 154 |
| Syria | 1 |
| Yemen | 2 |

**S1 Table. Geographic origin of sequences used in phylogenetic analysis.** Number of sequences African and Middle Eastern countries that were used in the MCC tree of HCV core isolates from Saudi Arabia.
