# Supplementary material for: Molecular characterization and epidemic history of hepatitis C virus using core sequences of isolates from Central Province, Saudi Arabia
Source: PLoS One. 2017 Sep 1;12(9):e0184163. doi: 10.1371/journal.pone.0184163 (PMC5580995; doi:10.1371/journal.pone.0184163)
Supplement: S2 Table — All references express HCV complete genomes and were chosen according to the subtype and country of origin. (DOCX) [file pone.0184163.s002.docx]

| **Country** | **References** | **Subtype** |
| --- | --- | --- |
| **Egypt** | DQ988074  Y11604 | 4a  4a |
| **USA** | DQ418789  DQ418786  EU392172  EU781760  HQ850279  EU718827  EU718828  EU718839  GU133617  FJ478453 | 4a  4d  4d  1a  1a  1b  1b  1b  1b  1b |

**S2 Table. List of references used in the construction of Maximum Likelihood trees and phylogenetic analysis of both HCV Core region.** All references express HCV complete genomes and were chosen according to the subtype and country of origin.

| **Country** | **References** | **Subtype** |
| --- | --- | --- |
| **Canada** | JF735737  FJ462437  FJ462433  Fj462441  FJ462439 | 4a  4d  4m  4n  4r |
| **Japan** | AB795432  AF207761 | 4a  1b |
| **UK** | Jx227972  JX227970  JX227977  JX227979  JX227976 | 4m  4n  4o  4o  4r |
